# Profiling transcriptomes of human SH-SY5Y neuroblastoma cells exposed to maleic acid

**DOI:** 10.7717/peerj.3175

**Published:** 2017-04-05

**Authors:** Chia-Chi Wang, Yin-Chi Lin, Yin-Hua Cheng, Chun-Wei Tung

**Affiliations:** 1 Ph.D. Program in Toxicology, College of Pharmacy, Kaohsiung Medical University, Kaohsiung City, Taiwan; 2 National Institute of Environmental Health Sciences, National Health Research Institutes, Miaoli County, Taiwan; 3 School of Pharmacy, College of Pharmacy, Kaohsiung Medical University, Kaohsiung City, Taiwan; 4 Institute of Environmental Engineering, National Sun Yat-sen University, Kaohsiung City, Taiwan; 5 Research Center for Environmental Medicine, Kaohsiung Medical University, Kaohsiung City, Taiwan

**Keywords:** Maleic acid, Toxicogenomics, Deferentially expressed gene, Intracellular calcium, Transcriptome

## Abstract

**Background:**

Maleic acid is a multi-functional chemical widely used in the field of industrial chemistry for producing food additives and food contact materials. As maleic acid may contaminate food by the release from food packages or intentional addition, it raises the concern about the effects of excessive dietary exposure to maleic acid on human health. However, the influence of maleic acid on human health has not been thoroughly studied. In silico toxicogenomics approaches have found the association between maleic acid and nervous system disease in human. The aim of this study is to experimentally explore the effects of maleic acid on human neuronal cells.

**Methods:**

A microarray-based transcriptome profiling was performed to offer a better understanding of the effects of maleic acid on human health. Gene expression profiles of human neuroblastoma SH-SY5Y cells exposed to three concentrations of maleic acid (10, 50, and 100 μM) for 24 h were analyzed. Genes which were differentially expressed in dose-dependent manners were identified and further analyzed with an enrichment analysis. The expression profile of selected genes related to the inferred functional changes was validated using quantitative polymerase chain reaction (qPCR). Specific fluorescence probes were applied to observe the inferred functional changes in maleic acid-treated neuronal cells.

**Results:**

A total of 316 differentially expressed genes (141 upregulated and 175 downregulated) were identified in response to the treatment of maleic acid. The enrichment analysis showed that DNA binding and metal ion binding were the significant molecular functions (MFs) of the neuronal cells affected by maleic acid. Maleic acid exposure decreased the expression of genes associated with calcium and thiol levels of the cells in a dose-dependent manner. The levels of intracellular calcium and thiol levels were also affected by maleic acid dose-dependent.

**Discussion:**

The exposure to maleic acid is found to decrease the cellular calcium and thiol levels in human neuronal cells at both transcriptional and functional levels. This study reported the first transcriptomic profiling of human neuronal cells treated with maleic acid. It is also the first experimental validation of chemical effects predicted by in silico toxicogenomics approaches. The proposed approach may be useful in understanding the potential effects of other poorly characterized chemicals on human health.

## Background

Maleic anhydride is readily converted into maleic acid in the presence of water. Both of maleic acid and maleic anhydride are multi-functional chemicals widely used in industrial chemical reactions, including the manufacture of alkyd resins, surface coatings, lubricants additives, plasticizers, copolymers, and agricultural chemicals (Felthouse et al., 2001, 2005; Cosmetic Ingredient Review Expert Panel, 2007). Maleic acid is also adopted as a precursor for producing food additives and may contaminate food as an impurity (Felthouse et al., 2001). The addition of maleic anhydride into modified starches has been found to enhance textures of starch-containing food products. However, the intentional addition of maleic acid and maleic anhydride has not been approved by major governmental authorities worldwide (DOH to Determine Source of Maleic Acid in Food, 2013). Given its current and anticipated future applications in industry, the effects of excessive exposure to maleic acid on human health are of great interest.

The effects of maleic acid on human health are poorly understood and mainly based on the results extrapolated from experimental animal data. The most well-known toxicity of maleic acid is nephrotoxicity in experimental animals fed with high doses of maleic acid. Maleic acid has been demonstrated to be negative to induce genotoxicity as well as reproductive and developmental toxicity with relatively low oral acute toxicity according to the current experimental animal data (Clayton & Clayton, 1981; Verani et al., 1982; Al-Bander et al., 1986; Cosmetic Ingredient Review Expert Panel, 2007). Our group has developed toxicogenomics approaches to systematically analyze the affected functions, pathways, and diseases associated with maleic acid based on chemical–protein interaction data (Lin, Wang & Tung, 2014; Tung, 2015). In addition to the known associated renal, behavioral, and gastrointestinal diseases (BIOFAX, 1970; Cosmetic Ingredient Review Expert Panel, 2007), the analyses found the new associations between maleic acid and diseases of mental health, nervous system, cardiovascular, and metabolism. Among these newly inferred diseases, the disease of nervous systems is of special interest due to the consistent analysis results of the affected pathway in the neuronal system (Lin, Wang & Tung, 2014; Tung, 2015). The effects of maleic acid on the nervous systems have been simply reported to affect seizure threshold in rats after oral uptake of a high dose of maleic acid (BIOFAX, 1970). Nevertheless, the underlying mechanism of maleic acid-mediated toxic effects on human neuronal system is still unknown.

In order to clearly understand the effects of maleic acid on neuronal functions and confirm the usefulness of using toxicogenomics approaches into inferring the effects of chemicals in human, the aim of the study is to evaluate the influence of maleic acid on neuronal functions by analyzing gene expression profiles of human SH-SY5Y neuroblastoma cells exposed to maleic acid. A total of 243 genes were identified to be differentially expressed with a dose–response relationship. An enrichment analysis on affected functions showed a consistent result with our previous analyses that metal ion binding could be disturbed by maleic acid. The treatment of maleic acid resulted in an attenuation of cellular calcium and thiol levels on SH-SY5Y cells without significant changes in cell viability. The expression levels of selected genes associated with calcium binding further confirmed the association. This study represents the first transcriptomics report on the effects of maleic acid on human neuronal cells as well as the first experimental evidence that the in silico inference from ChemDIS system is useful in understanding potential effects of chemicals on human health.

## Materials and Methods

### Reagents

All reagents were purchased from Sigma (St. Louis, MO, USA) unless otherwise stated.

### Cell lines and culture conditions

The human neuroblastoma cell line, SH-SY5Y, was purchased from ATCC (Manassas, VA, USA). Cells were cultured in a 1:1 mixture of ATCC-formulated Eagle’s minimum essential medium and F12 medium supplemented with 2 mM l-glutamine, 1% of penicillin/streptomycin, and 10% heat-inactivated fetal bovine serum (GIBCO, Gaithersburg, MD, USA) at 37 °C in 5% CO_2_. Cells were seeded at an initial density of 10^4^ cells/cm^2^ and cultured overnight prior to the treatment with various concentrations of maleic acid (Sigma-Aldrich, St. Louis, MO).

### Cell viability

Cell viability was evaluated by 3-(4,5-dimethylthiazol-2-yl)-5-(3-carboxymethoxyphenyl)-2-((4-sulfophenyl)-2*H*-tetrazolium) chloride (MTS) assay (The CellTiter 96® AQ_ueous_ One Solution Cell Proliferation Assay kit) according to the manufacturer’s instruction. Briefly, cells (4 × 10^4^) were seeded in a 96-well plate overnight and then exposed to maleic acid for 24 and 48 h. MTS solution was added to each well for 4 h at 37 °C and the absorbance was read at wavelength 490 nm by a microplate reader (Thermo Varioskan Flash, Thermo, Winooski, VT, USA).

### Measurement of intracellular calcium

Fluo-4 AM, a membrane-permeable Ca^2+^-sensitive fluorescent dye, was used to measure the free cytosolic calcium. Cells were seeded at 2 × 10^4^ cells/well in 96-well clear-bottomed black microplates (Corning Costar, Tewksbury, MA, USA) for overnight. Cells were treated with serial concentrations of maleic acid and the supernatants of each well were removed. Next, the cells were washed with PBS twice and incubated with 5 μM of Fluo-4 AM (ThemoFisher) at 37 °C for 15 min. The change in fluorescence at excitation/emission of 494/506 nm was analyzed by a microplate reader (Thermo Varioskan Flash, Thermo, USA).

### Measurement of intracellular thiols

The depletion of intracellular thiols in maleic acid-treated cells was determined by CMF-DA staining. SH-SY5Y cells were seeded at 2 × 10^4^ cells/well in 96-well clear-bottomed black microplates overnight. Then, the culture medium was replaced with new medium containing maleic acid (0.1–100 μM) for 1, 8, and 24 h. After treatment, cells were washed with PBS and then stained with 25 μM of CMF-DA for 30 min at 37 °C. Then, the cells were washed twice with PBS. The change in fluorescence at excitation/emission of 488/525 nm was analyzed by a microplate reader (Thermo Varioskan Flash, Thermo, USA).

### Total RNA isolation

Total RNA was extracted from SH-SY5Y cells using the TRIzol (Qiagen, Valencia, CA, USA). The RNA samples were then treated with an RQ1 RNase-free DNase kit (Promega, Southampton, UK) according to the manufacturer’s instructions to remove contaminated DNA. The RNA concentration and purity were checked by OD260/OD280 (>1.8) and OD260/OD230 (>1.6), and the yield and quality were assessed using Agilent 2100 Bioanalyzer (Agilent Technologies, Santa Clara, CA, USA).

### Human oligonucleotide DNA microarray

The Human Whole Genome OneArray® v6 (Phalanx Biotech Group, Hsinchu, Taiwan) contains 32,679 DNA oligonucleotide probes, and each probe is a 60-mer designed in the sense direction. Among the probes, 31,741 probes correspond to the annotated genes in RefSeq v51 and Ensembl v65 database. Besides, 938 control probes are also included. The detailed descriptions of the gene array are available from https://www.ncbi.nlm.nih.gov/geo/query/acc.cgi?acc=GPL19137.

### Microarray analysis

Fluorescent antisense RNA (aRNA) targets were prepared from 1 μg total RNA samples using OneArray® Amino Allyl aRNA Amplification Kit (Phalanx Biotech Group, Taiwan) and Cy5 dyes (Amersham Pharmacia, Piscataway, NJ, USA). Fluorescent targets were hybridized to the Human Whole Genome OneArray® with phalanx hybridization buffer using phalanx hybridization system. After hybridization at 50 °C for 16 h, non-specific binding targets were washed out three times. First, the slides were washed twice at 42 °C for 5 min followed by another wash at 25 °C for 5 min. The slides were then rinsed for 20 times and dried by centrifugation. Finally, the slides were scanned via an Agilent G2505C scanner (Agilent Technologies, Santa Clara, CA, USA). The Cy5 fluorescent intensities of each spot were analyzed by GenePix 4.1 software (Molecular Devices, Sunnyvale, CA, USA). The signal intensity of each spot was processed by Rosetta Resolver System® (Rosetta Biosoftware, Seattle, WA, USA). The error model of Rosetta Resolver System® could provide a judgment of the probe signal’s credibility by removing both systematic and random errors within the data. Unreliable spots with a flag less than zero were filtered out. Spots that passed the criteria were normalized by 50% median-scaling normalization method. The technical repeat data was tested by Pearson correlation coefficients to check the reproducibility (*r* > 0.975). Normalized spot intensities were transformed into gene expression log_2_ ratios between the control and treatment groups. The data discussed in this study have been deposited in NCBI’s Gene Expression Omnibus (Edgar, Domrachev & Lash, 2002) and are accessible through GEO series accession number GSE86510.

**Figure 1:**
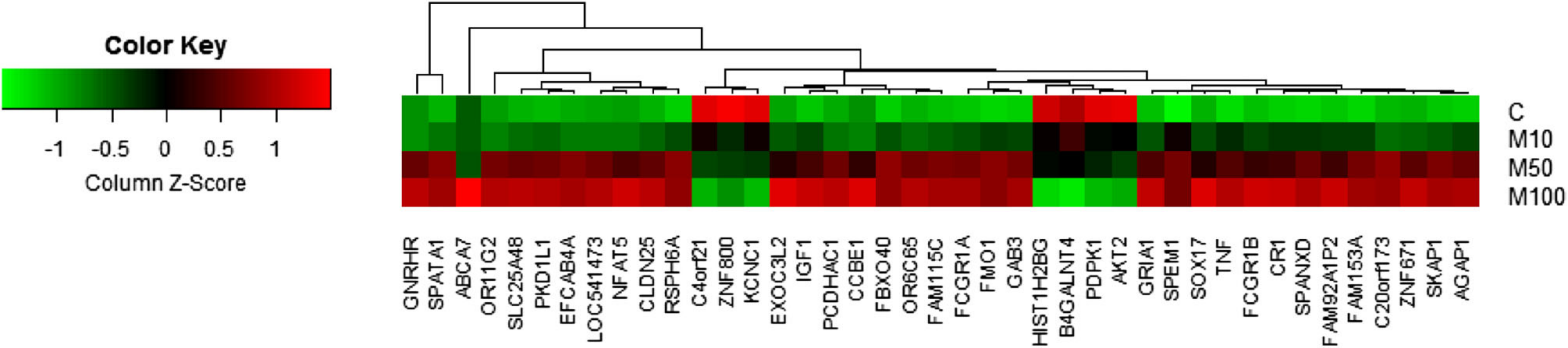
Heatmap of genes with a dose-dependent trend. Each spot represents the average value of technical replicates.

For the identification of differentially expressed genes, fold changes of probes are first calculated based on the ratio error model from Rosetta Resolver System® (Weng et al., 2006). Probes with a fold change larger than two are considered as differentially expressed genes. Compared to the conventional *t*-test, the error model helps to reduce false positives in comparison of gene expressions in two treatments with multiple replicates. In addition to the identification of differentially expressed genes based on the Rosetta error model, the average values of technical replicates were also calculated for creating a heatmap for visualization as shown in Fig. 1.

### Enrichment analysis

Enrichment-analysis tools were implemented and integrated into ChemDIS (Tung, 2015) in order to analyze functions, pathways, and diseases from a given set of differentially expressed genes. For enriched functions, clusterProfiler (Yu et al., 2012) providing comprehensive enrichment analysis functionalities was applied to analyze enriched gene ontology (GO) terms for MF, biological process (BP), and cellular component (CC). The enriched KEGG (Kanehisa et al., 2014) and Reactome (Croft et al., 2014) pathways were analyzed using clusterProfiler and ReactomePA (Yu & He, 2016), respectively. For inferring diseases affected by a given chemical, enriched DO (Kibbe et al., 2015), and DOLite (Du et al., 2009) terms will be analyzed using DOSE package (Yu et al., 2015). DOLite is a simplified vocabulary list of DO, a standardized ontology connecting human proteins to diseases. All the enrichment analyses are based on hypergeometric tests with the Benjamini–Hochberg approach (Du et al., 2009) for multiple-testing corrections. Enriched terms with a corrected *p*-value <0.05 will be identified. The enrichment analysis function of ChemDIS is freely available at http://cwtung.kmu.edutw/chemdis and the analysis in this study is reproducible using the service.

### Real-time reverse transcription-polymerase chain reactions

The concentration of RNA was determined by measuring its absorbance at 260 nm with a microplate reader (Thermo Varioskan Flash, Thermo, USA). Two micrograms of total RNA collected from each sample were reverse-transcribed through RevertAid RT Kit (Thermo) into cDNA products using oligo-dT as the primer. Real-time polymerase chain reaction (PCR) was performed in a 96-well optical tray by an ABI PRISM® 7900HT Sequence Detection System (Applied Biosystems, Cheshire, UK). During the real-time reverse transcription-polymerase chain reaction (RT-PCR) process, we used Luminaris Color HiGreen High ROX qPCR Master Mix (Thermo Fisher Scientific, Waltham, MA, USA) which offered a highly specific and sensitive method to quantify mRNA expressions. The HPRT gene was used as an endogenous control to normalize the expressions of target genes. The primers are: 5′-GTTCTGGACACCAACAAGGAC3′ and 5′-TCGTGGCAGTAGAGACA-GAGG-3′ for S100A3, 5′-AACTACTCTGGCAGGATCTGGA-3′ and 5′-AGGGGG-TTAGGGATGAGGCT-3′ for GGT5, 5′-GCTAAAGGTTTTGGAGCCCAC-3′ and 5′-AGAGTGGAGAGACATAGAGATGGT-3′ for GRIA1, 5′-GTTCTCCTTCATCCTATTTATGTGG-3′ and 5′-CCCTGTCCAAATCCTAACCCC-3′ for GNRHR, and 5′-TCAGTCAACGGGGGACATAAA-3′ and 5′-GGGGCTGTACTGCTTAACCAG-3′ for HPRT.

### Statistical analysis

The data of cell viability, cellular levels of calcium and thiols, and RNA expression were presented as mean values and standard errors. One-way analysis of variance was adopted for multiple comparisons, and Dunnett’s two-tailed *t*-test was used to compare the results of the treatment groups with those of the control group using JMP statistical software (Cary, NC, USA). *p* < 0.05 was defined as statistically significant.

## Results and Discussion

### Transcriptome profiling and differentially expressed genes

To understand more about the effects of maleic acid on neuronal cells, the gene expression profiles of SH-SY5Y cells treated with three doses (10, 50, and 100 μM) of maleic acid were examined. The assessment of variability, which comes from biological and technical replicates, is an important concern for microarray experiments. However, a high intra-platform consistency of microarray experiments for both technical and biological replicates has been observed with correlation coefficients higher than 0.9 (Tan et al., 2003). Compound-induced changes have also been shown to outweigh the influence of the technical and biological variability in a microarray experiment (Bryant et al., 2011).

To minimize the selection bias caused by biological variability, this study adopted a scheme of three treatments with different doses in the experimental group versus one control, and only differentially expressed genes with monotonically increased or decreased expression values were considered for the following analysis. In addition, two technical replicates of each sample were utilized to confirm the reproducibility. The monotonically increased/decreased genes were identified by applying simple filtering rules. Given a candidate gene with four expression values, the following rule was applied to define a monotonically increased gene. IF (*H* > *M*) AND (*M* > *L*) AND (*L* > *N*) THEN the gene is a monotonically increased genes, where *H*, *M*, *L*, and *N* represent its expression values for the treatments of maleic acid in different doses (100, 50, 10, and 0 μM), respectively.

A total of 389 differentially expressed probes (191 upregulated and 198 downregulated) were identified with more than twofold changes over control after applying the highest dose (100 μM) of maleic acid (Table S1). To remove probes without corresponding gene annotations, 46 and 9 probes with keywords of “NA” and “uncharacterized,” respectively, in the corresponding description field were excluded from subsequent analysis. Finally, 243 differentially expressed genes were identified (113 upregulated and 130 downregulated). Detail information for the 243 differentially expressed genes is available in Table S1. Among the 243 differentially expressed genes identified by the Rosetta error model (Weng et al., 2006), 48 genes were also found to have a clear dose-dependent trend in response to maleic acid treatment based on the average expression values from technical replicates. Figure 1 shows the heatmap of the 48 genes, including 42 upregulated and six downregulated genes.

### Enrichment analysis

**Figure 2:**
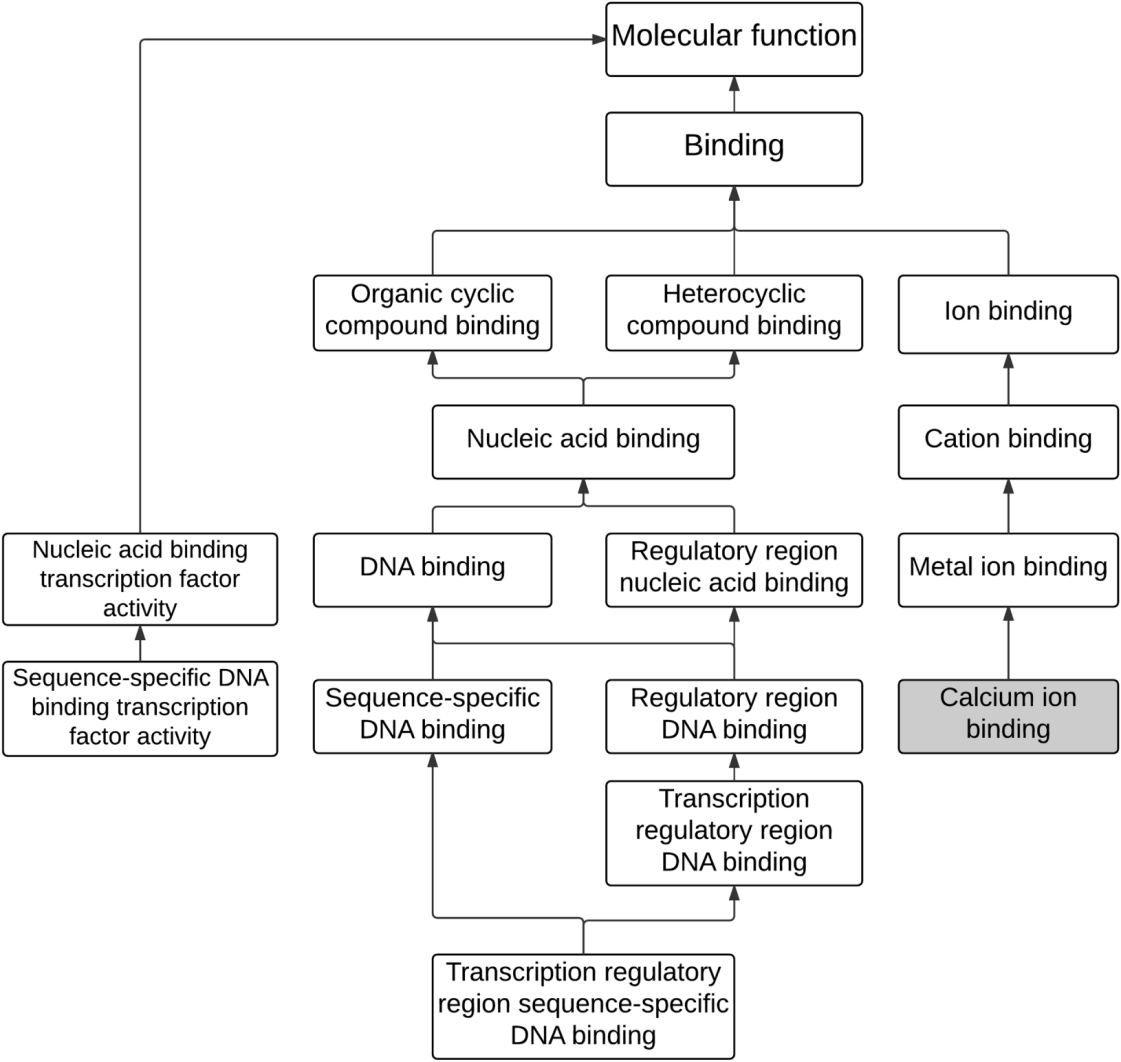
The hierarchical tree of enriched GO terms associated with molecular functions.

The analysis of enriched GO terms was conducted for the 243 differential expressed genes to identify affected functions associated with maleic acid. As shown in Table 1, there were 12 significantly enriched GO terms (corrected *p*-value <0.05), including eight MFs, two CCs, and two BPs. The enriched MF GO terms were visually analyzed as shown in Fig. 2. Two main branches with hierarchy levels higher than binding (GO:005488) were identified. Metal ion binding (GO:0046872), cation binding (GO:0043169), and ion binding (GO:0043167) are located at the same branch, whereas DNA binding (GO:003677), nucleic acid binding (GO:0003676), heterocyclic compound binding (GO:1901363), and organic cyclic compound binding (GO:0097159) are located at the other branch. Both branches of GO terms of metal ion binding and DNA binding were consistent with our previous analysis from ChemDIS system (Lin, Wang & Tung, 2014; Tung, 2015). The calcium binding (GO:0005509), a direct descendant of the enriched term of metal ion binding (GO:0046872), was inferred to be associated with maleic acid. The enriched GO term of DNA binding (GO:0003677) is related to nucleotide binding (GO:0000166) that was inferred to be associated with maleic acid from ChemDIS (Lin, Wang & Tung, 2014; Tung, 2015). The enriched pathways are hematopoietic cell lineage (KEGG ID:hsa04640) and generic transcription pathway (Reactome ID: 212436), and there are no enriched DO and DOLite terms from the analysis. For validating the enrichment analysis in this study, the same gene set was applied to analyze enriched terms using the comparative toxicogenomics (CTD) database. As expected, except for the analysis of enriched disease terms, ChemDIS and CTD generated similar enriched GO and pathway terms as shown in Table S2. ChemDIS and CTD utilizing different disease ontologies could generate different results of enriched diseases (Tung, 2015).

**Table 1:** Enriched GO terms associated with molecular functions.

Type	GO ID	Description	*p*-value	Corrected *p*-value	Number of genes
MF	GO:0005488	Binding	6.45E−08	3.19E−06	207
MF	GO:0003677	DNA binding	1.55E−07	5.10E−06	62
MF	GO:0043167	Ion binding	2.75E−06	6.80E−05	115
MF	GO:0003700	Sequence-specific DNA binding transcription factor activity	5.17E−06	7.70E−05	33
MF	GO:0001071	Nucleic acid binding transcription factor activity	5.28E−06	7.70E−05	33
MF	GO:0003676	Nucleic acid binding	5.45E−06	7.70E−05	82
MF	GO:1901363	Heterocyclic compound binding	1.32E−05	1.64E−04	109
MF	GO:0097159	Organic cyclic compound binding	2.36E−05	2.60E−04	109
MF	GO:0043169	Cation binding	3.32E−05	3.29E−04	83
MF	GO:0046872	Metal ion binding	5.67E−05	5.11E−04	81
MF	GO:0002020	Protease binding	5.77E−04	4.76E−03	6
MF	GO:0043565	Sequence-specific DNA binding	1.39E−03	1.05E−02	21
MF	GO:0044212	Transcription regulatory region DNA binding	3.84E−03	2.70E−02	15
MF	GO:0000975	Regulatory region DNA binding	4.36E−03	2.70E−02	15
MF	GO:0001067	Regulatory region nucleic acid binding	4.36E−03	2.70E−02	15
MF	GO:0032403	Protein complex binding	5.34E−03	3.11E−02	20
MF	GO:0000976	Transcription regulatory region sequence-specific DNA binding	5.78E−03	3.18E−02	11

### Attenuation of cellular calcium and thiol levels by maleic acid

**Figure 3:**
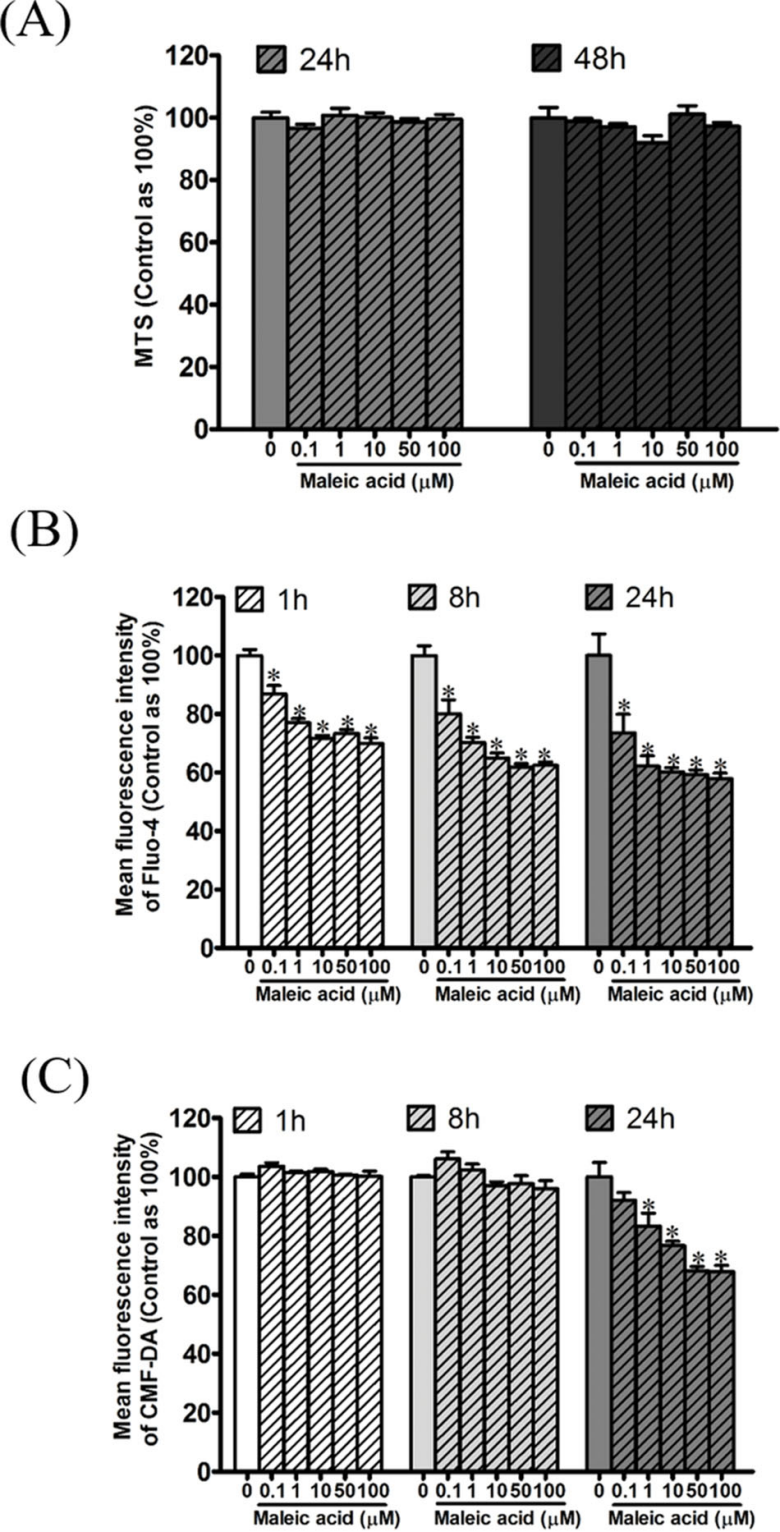
Attenuation of cellular calcium and thiol level by maleic acid in SH-SY5Y cells. SH-SY5Y cells were seeded in a 96-well plate overnight and then exposed to maleic acid (0.1–100 μM). (A) Cell viability was evaluated by MTS and the absorbance was read at wavelength 490 nm by a microplate reader. Intracellular calcium and thiols of SH-SY5Y cells were measured by Fluo-4 AM (B) and CMF-DA (C), respectively. The change in fluorescence was analyzed by a microplate reader. The data were expressed as the mean ± SE of quadruplicate cultures. Results were representative of three independent experiments. *p < 0.05 was significant compared to the control group (cell alone without maleic acid).

According to our previous transcriptome and enrichment analysis, calcium binding was inferred to be affected by maleic acid. Among the differentially expressed genes, eight genes are associated with the GO term of calcium binding (GO:0005509) including S100A3, GNPTAB, CDH23, PCDHAC1, PADI1, CRACR2B, MAN1A2, and CDH8. In addition, GNRHR and GRIA1 among the differentially expressed genes are involved in the regulation of cellular calcium levels. Altogether, cellular calcium is potentially affected by maleic acid. To validate this finding, the kinetic change of cellular calcium and thiol levels by maleic acid was analyzed by specific fluorescence Fluo-4 and CMF-DA probes, respectively. To avoid measuring the direct cytotoxic effects of maleic acid on SH-SY5Y cells, we first evaluated the cell viability of maleic acid-treated SH-SY5Y cells by an MTS assay. Figure 3A showed that there was no significant change in cell viability after 48 h of maleic acid treatment. By contrast, maleic acid (0.1–100 μM) significantly decreased the cellular calcium level by 12–41%, determined by the change of fluorescence intensity of Fluo-4, in a concentration- and time-dependent manner (Fig. 3B). Maleic acid (1–100 μM) decreased the cellular thiol levels by 16–31% after 24 h treatment (Fig. 3C). The exact *p*-values of Fig. 3 were shown in Supplemental Information 2. These results indicated that maleic acid resulted in a loss of cellular calcium level earlier than glutathione depletion. These observations could give the evidence that maleic acid at the concentrations higher than 0.1 μM interferes cellular calcium homeostasis in human neuronal cells. The homeostasis of intracellular Ca^2+^ plays important roles in the regulation of physiological responses or controlling cell death (Demuro et al., 2005; Bading, 2013; Brini et al., 2014). Depletion of intracellular Ca^2+^ has been reported to induce SH-SY5Y cell death by activating caspase activation (McGinnis, Wang & Gnegy, 1999). Our data showed that maleic acid significantly attenuated intracellular Ca^2+^ levels without induction of cell death, indicating that other pathways might be involved and need to be further studied.

### qPCR validation

According to our transcriptome analysis, there were 243 genes whose expression were significantly affected by maleic acid in a dose-dependent manner. To confirm the association between the gene regulation and the attenuated cellular calcium and thiol levels by maleic acid, we evaluated the expression of GGT5, GNRHR, GRIA1, and S100A3 by real-time qPCR using three independent biological replicates to further prove the reliability of the microarray data.

**Figure 4:**
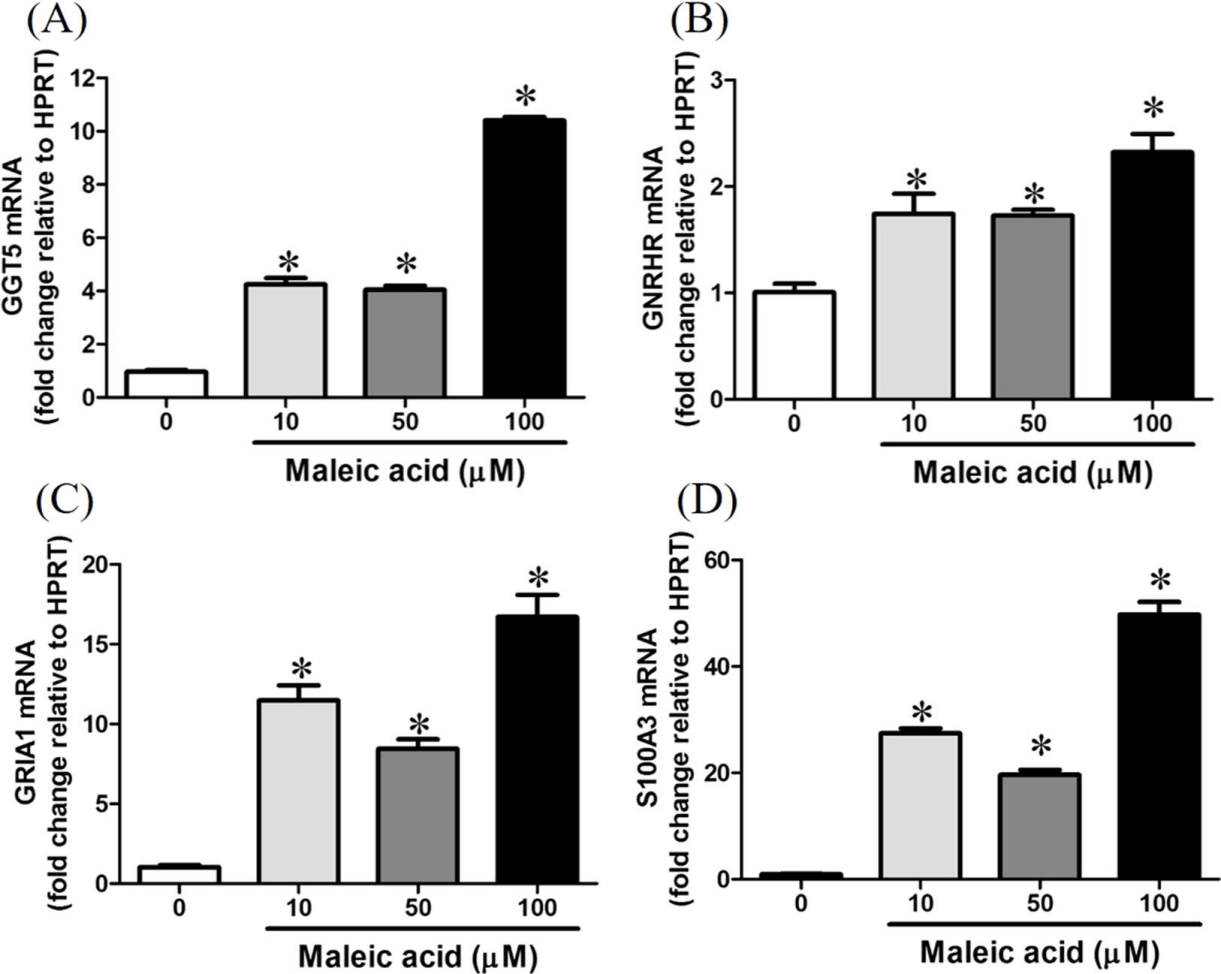
Increasing of GGT5, GNRHR, GRIA1, and S100A3 gene expression by maleic acid. SH-SY5Y cells were treated with maleic acid (10–100 μM) for 24 h. The total RNA of cells was extracted and the mRNA expression of (A) GGT5, (B) GNRHR, (C) GRIA1, and (D) S100A3 was measured by real-time RT-PCR. The expression level of HPRT was used as the control for semi-quantification. Results were expressed as the mean ± SE of pooled data from three independent experiments. *p < 0.05 was significant compared to the control group (cell alone without maleic acid).

Maleic acid upregulated the expression of GGT5 in a dose-dependent manner (Fig. 4A). Gamma-glutamyl transferase (GGT) family genes encode key enzymes widely distributed in various tissues in response to different stimuli (Lieberman et al., 1995). They might play important roles in glutathione salvage, detoxification of xenobiotics, and metabolism of endogenous mediators such as prostaglandins and leukotrienes (Lieberman et al., 1995). In neurons, GGT enzymes liberate glutamate from glutathione. Glutathione has been suggested as a reservoir of neuronal glutamate (Koga et al., 2011), which is the main excitatory transmitter in the central nervous system to participate in many important physiological processes including long-term potentiation and developmental plasticity (McEntee & Crook, 1993). Deficiency of glutathione has been reported in several neuropsychiatric disorders (Gu, Chauhan & Chauhan, 2015). Dysregulation of GGT may interfere with the glutathione synthesis and cellular glutamate levels.

Maleic acid also upregulated GRIA1, GNRHR, and S100A3 in a dose-dependent manner (Figs. 4B–4D). The exact *p*-values of Fig. 4 were shown in Supplemental Information 2. GRIA1, GNRHR, and S100A3 are associated with calcium influx or binding. GRIA1 encodes glutamate receptor 1, which is a subunit of calcium-permeable α-amino-3-hydroxy-5-methyl-4-isoxazolepropionate (AMPA) glutamate receptors. Glutamate receptors are activated in a variety of normal neurophysiologic processes and are responsible for the glutamate-mediated postsynaptic excitation of neuronal cells. GNRHR gene encodes gonadotropin-releasing hormone receptor, which belongs to seven-transmembrane G-protein coupled receptor (Millar, 2005). Activation of GNRHR will further activate a phosphatidylinositol-calcium second messenger system and further regulate the release of follicle-stimulating hormone and luteinizing hormone (Romanelli et al., 2004). S100A3 encodes S100 calcium-binding protein A3 which contains two EF-hand calcium-binding motifs and plays roles in cell cycle progression and differentiation (Schäfer & Heizmann, 1996). The increased expression of S100A3 has been associated with methamphetamine-mediated neuronal toxicity (Cadet et al., 2011). As GNRHR, GRIA1, and S100A3 were all significantly augmented by maleic acid, the finding suggested that the dysregulation of these genes may participate in maleic acid-mediated toxicity.

Collectively, the present data demonstrated that maleic acid interfered with the expression level of genes associated with calcium influx or binding and further gave the mechanical evidence on how maleic acid affects human neuronal cells. However, the alteration of functions of the neuronal cells by these proteins in response to maleic acid treatment was not evaluated in this study and warrant further study. Nonetheless, the study has set an example of utilizing ChemDIS system to prioritize the potential effects of maleic acid on the human health and to generate testable experimental approaches which could validate the inferred association. This approach might also be useful for understanding other poorly characterized chemicals.

## Conclusion

This study shows the effects of maleic acid on human SH-SY5Y neuronal cells by profiling and analyzing the transcriptomes. The enrichment analysis of 243 differentially expressed genes identifies two functions affected by maleic acid including DNA binding and metal ion binding. The attenuation of calcium and thiol levels in SH-SY5Y cells indicates that the maleic acid significantly interferes cellular calcium homeostasis and induces the depletion of cellular thiols. The upregulated expression of GRIA1, GNRHR, and S100A3 may be involved in the dysregulation of calcium homeostasis in maleic acid-treated neuronal cells. These experimental results are consistent with the analyses from the ChemDIS system. The workflow incorporating in silico and in vitro analyses could be useful for exploring the potential effects of poorly characterized chemicals on human health.

## Supplemental Information

10.7717/peerj.3175/supp-1 Supplemental Information 1 Raw data for Fig. 1.

10.7717/peerj.3175/supp-2 Supplemental Information 2 Raw data for Figs. 3 and 4.

10.7717/peerj.3175/supp-3 Supplemental Information 3 MIAME.

10.7717/peerj.3175/supp-4 Supplemental Information 4 Differentially expressed genes.

10.7717/peerj.3175/supp-5 Supplemental Information 5 A comparison of enrichment analysis from ChemDIS and CTD.

## Additional Information and Declarations

### Microarray Data Deposition

The following information was supplied regarding the deposition of microarray data: GSE86510.

### Data Availability

The following information was supplied regarding data availability: The raw data has been supplied as Supplemental Dataset Files.
