# Supplementary material for: Profiling transcriptomes of human SH-SY5Y neuroblastoma cells exposed to maleic acid
Source: PeerJ. 2017 Apr 5;5:e3175. doi: 10.7717/peerj.3175 (PMC5384566; doi:10.7717/peerj.3175)
Supplement: Supplemental Information 3 [file peerj-05-3175-s003.pdf]

Part 1 Experiment description

- **cell type**
  Human SH-SY5Y neuroblastoma cells
- **experimental variables**
  Control, Maleic acid (10, 50 and 100 μM)
- **n-count**
  Two technical repeats for each experimental variable

Part 2 Array design

The Human Whole Genome OneArray® v6 (Phalanx Biotech Group, Taiwan) contains 32,679 DNA oligonucleotide probes, and each probe is a 60-mer designed in the sense direction. Among the probes, 31,741 probes correspond to the annotated genes in RefSeq v51 and Ensembl v65 database. Besides, 938 control probes are also included. The detailed descriptions of the gene array are available from The detailed descriptions of the gene array are available from https://www.ncbi.nlm.nih.gov/geo/query/acc.cgi?acc=GPL19137.

Part 3 Samples

Fluorescent aRNA targets were prepared from 1 μg total RNA samples using OneArray® Amino Allyl aRNA Amplification Kit (Phalanx Biotech Group, Taiwan) and Cy5 dyes (Amersham Pharmacia, Piscataway, NJ, USA).

Part 4 Hybridizations

Fluorescent targets were hybridized to the Human Whole Genome OneArray® with Phalanx hybridization buffer using Phalanx Hybridization System. After hybridization at 50℃ for 16 h, non-specific binding targets were washed away through three different washing steps. First, the slides were washed at 42℃ for 5 min and then washed at 42℃ for 5 min followed by another wash at 25℃ for 5 min. In the final clean-up step, the slides were rinsed for 20 times.

Part 5 Measurements

Fluorescent antisense RNA (aRNA) targets were prepared from 1 μg total RNA samples using OneArray® Amino Allyl aRNA Amplification Kit (Phalanx Biotech Group, Taiwan) and

Cy5 dyes (Amersham Pharmacia, Piscataway, NJ, USA). Fluorescent targets were hybridized to the Human Whole Genome OneArray® with Phalanx hybridization buffer using Phalanx Hybridization System. After hybridization at 50℃ for 16 h, non-specific binding targets were washed out three times. First, the slides were washed twice at 42℃ for 5 min followed by another wash at 25℃ for 5 min. The slides were then rinsed for 20 times and dried by centrifugation. Finally, the slides were scanned via an Agilent G2505C scanner (Agilent Technologies, Santa Clara, CA, USA). The Cy5 fluorescent intensities of each spot were analyzed by GenePix 4.1 software (Molecular Devices, Sunnyvale, CA, USA). The signal intensity of each spot was processed by Rosetta Resolver System® (Rosetta Biosoftware, Seattle, WA, USA). The error model of Rosetta Resolver System® could provide a judgment of the probe signal's credibility by removing both systematic and random errors within the data. Unreliable spots with a flag less than 0 were filtered out. Spots that passed the criteria were normalized by 50% median scaling normalization method. The technical repeat data was tested by Pearson correlation coefficients to check the reproducibility ($r>0.975$). Normalized spot intensities were transformed into gene expression log2 ratios between the control and treatment groups. The data discussed in this study have been deposited in NCBI's Gene Expression Omnibus (Edgar, Domrachev & Lash, 2002) and are accessible through GEO Series accession number GSE86510 (http://www.ncbi.nlm.nih.gov/geo/query/acc.cgi?acc=GSE86510).

Part 6 Normalization controls

This study tried to identify genes differentially expressed in a dose-dependent manner, i.e., the genes with monotonically increased/decreased expressions resulted from increased doses of maleic acid compared with controls. Controls are human SH-SY5Y neuroblastoma cells without treatment of maleic acid. The probe set that passes the filtering condition will be normalized using 50% median scaling between array data sets. From the analysis, 316 differentially expressed genes (141 up- and 175 down-regulated).
